# Supplementary material for: A high capacity small molecule quinone cathode for rechargeable aqueous zinc-organic batteries
Source: Nat Commun. 2021 Jul 20;12:4424. doi: 10.1038/s41467-021-24701-9 (PMC8292436; doi:10.1038/s41467-021-24701-9)
Supplement: Supplementary file 1 — Supplementary Information [file 41467_2021_24701_MOESM1_ESM.pdf]

Supplementary Information

A High Capacity Small Molecule Quinone Cathode for Rechargeable Aqueous Zinc-Organic Batteries

Zirui Lin[1], Hua-Yu Shi[1], Lu Lin[1], Xianpeng Yang[1], Wanlong Wu[1], and Xiaoqi Sun[1,*]

[1]Department of Chemistry, Northeastern University, 3-11 Wenhua Road, Shenyang, 110819, China

*E-mail: sunxiaoqi@mail.neu.edu.cn

Supplementary Figures and Table

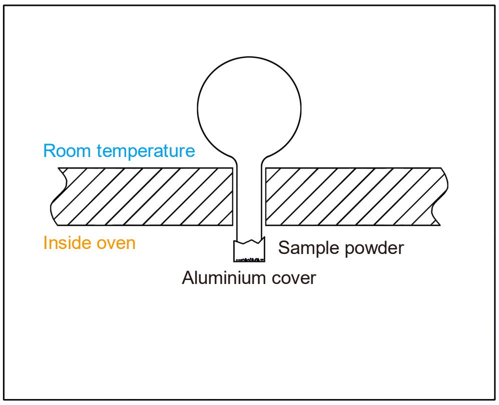

Supplementary Figure 1. Schematic illustration for the setup for sublimation behavior studies.

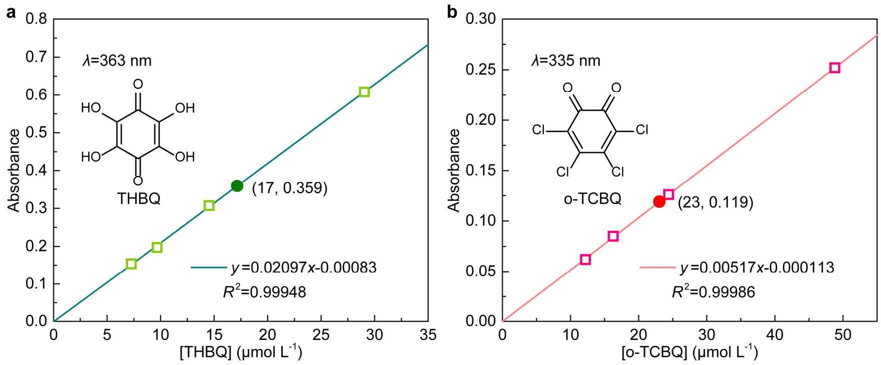

Supplementary Figure 2. UV-vis analysis to calculate the solubility of quinone materials: calibration curves of **a** THBQ and **b** o-TCBQ in 1 M $ZnSO_4$.

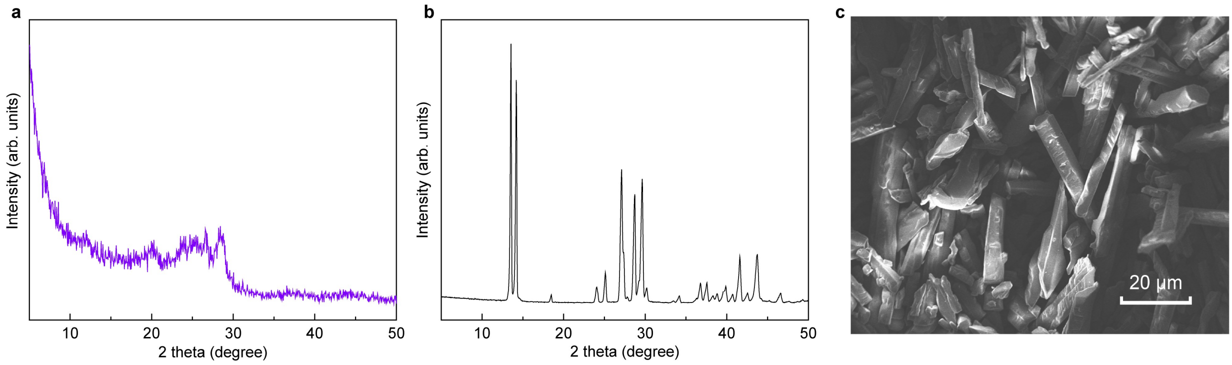

Supplementary Figure 3. **a** XRD of the TABQ electrode. **b** XRD and **c** SEM image of the as-prepared TABQ.

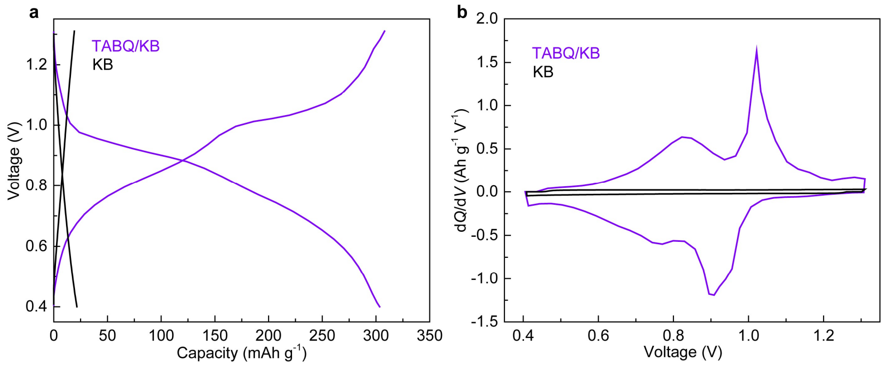

Supplementary Figure 4. Electrochemical performance of TABQ/KB and KB electrodes in 1 M $ZnSO_4$: **a** charge/discharge curves and **b** differential capacity curves.

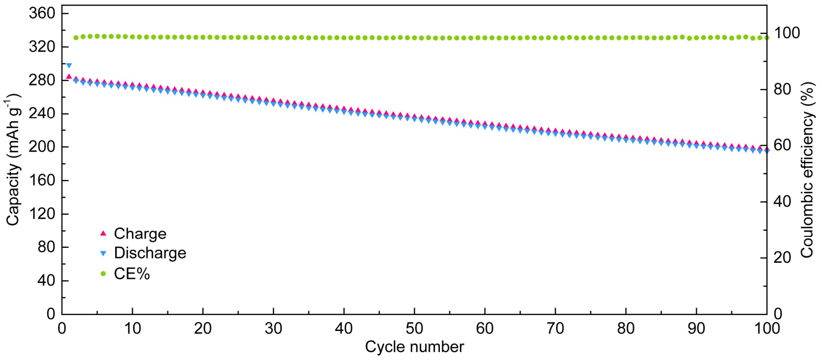

Supplementary Figure 5. Capacity and coulombic efficiency evolution of TABQ at 0.16 A g^{-1} for 100 cycles.

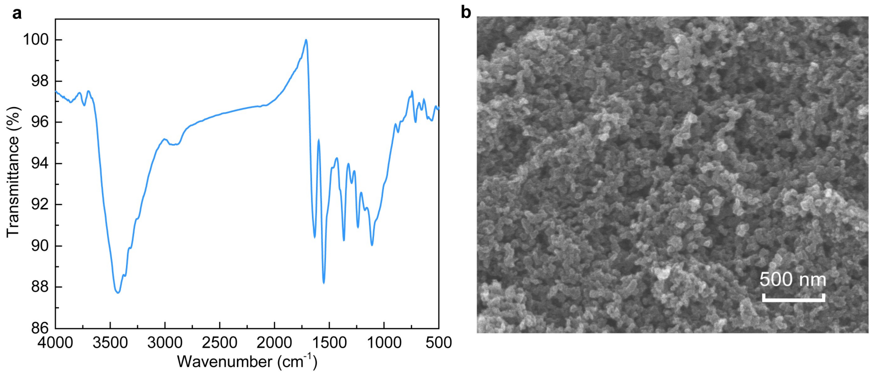

Supplementary Figure 6. Characterizations of the TABQ electrode after 1000 cycles: **a** FT-IR spectrum. **b** SEM image.

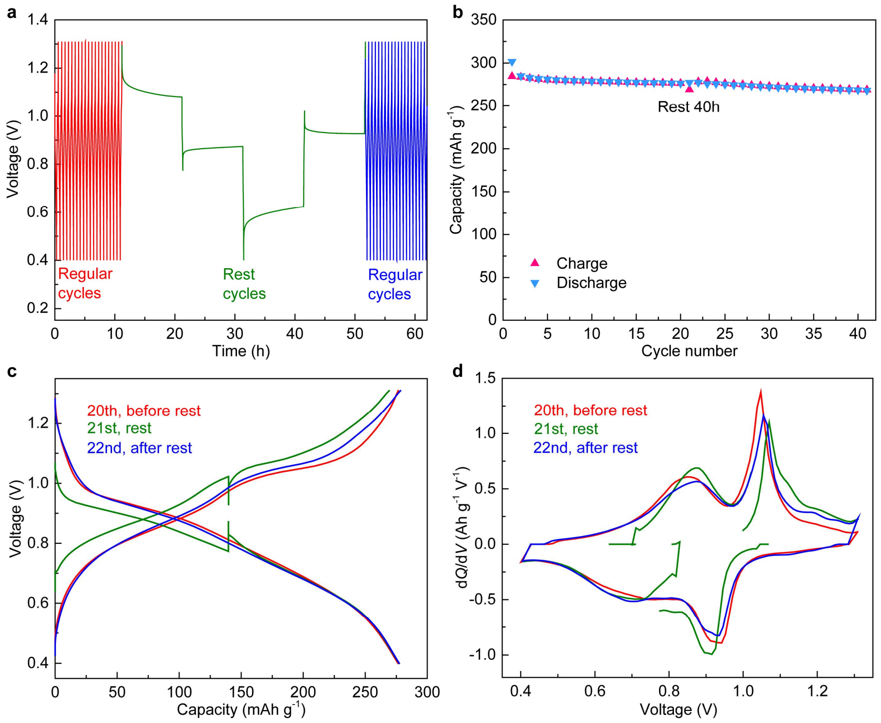

Supplementary Figure 7. Electrochemical performance of the TABQ cathode during rest test: **a** voltage evolution over time; **b** capacity evolution of TABQ; **c** charge/discharge curves and **d** differential capacity curves before, during and after the cycle with rest.

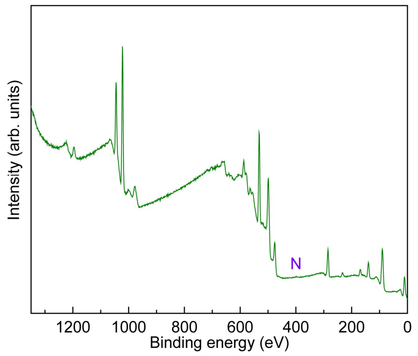

Supplementary Figure 8. XPS of the Zn anode after 10 cycles at 0.1 A g^{-1} in the cell with TABQ cathode and the electrolyte of 1 M $ZnSO_4$ containing saturated TABQ.

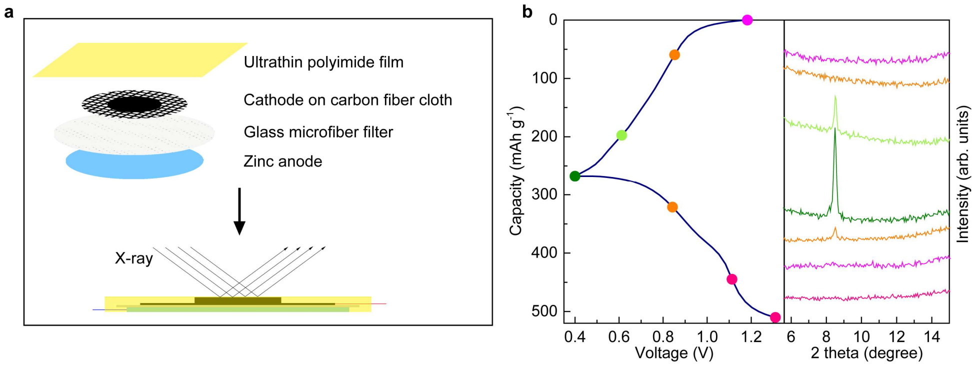

Supplementary Figure 9. In-situ XRD of TABQ: **a** schematic diagram of the in-situ XRD analysis; **b** XRD peak evolution along discharge and charge.

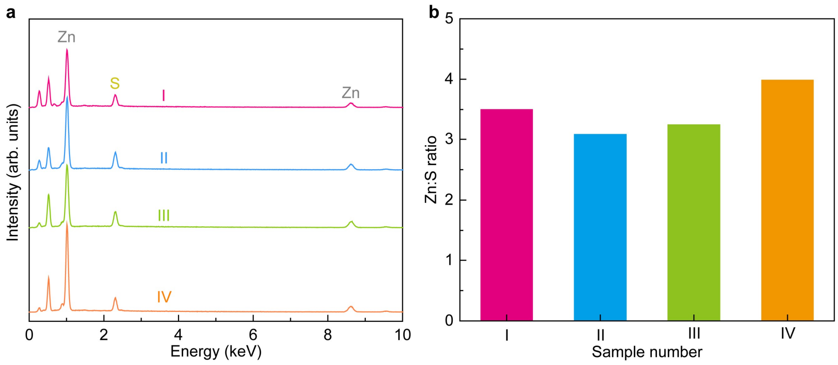

Supplementary Figure 10. EDS analysis of discharged TABQ: **a** EDS spectra and **b** the corresponding Zn:S molar ratios from four parallel measurements.

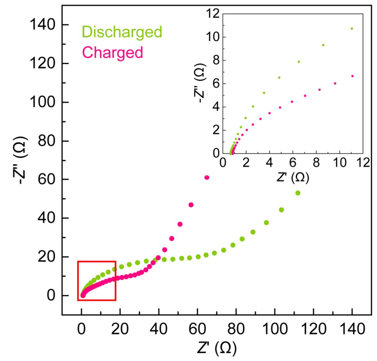

Supplementary Figure 11. The Nyquist plots of TABQ at the end of discharge and charge.

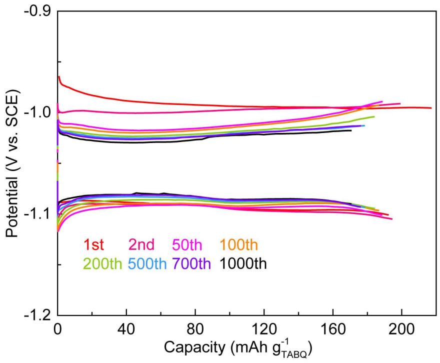

Supplementary Figure 12. Charge/discharge curves of Zn electrode during long-term cycling test.

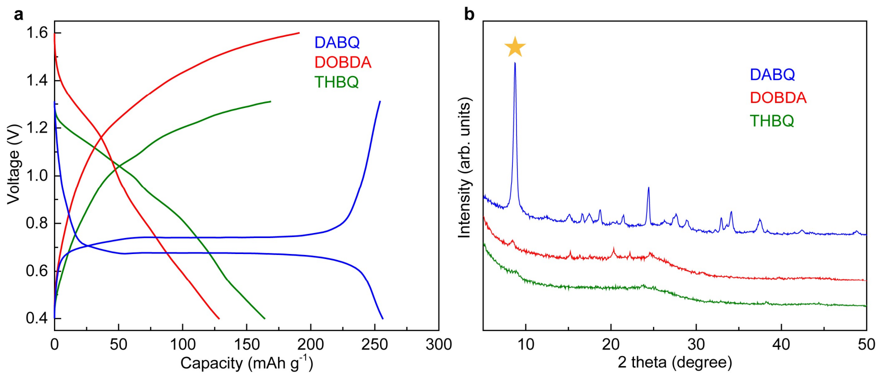

Supplementary Figure 13. Electrochemical performance of DOBDA, THBQ and DABQ in aqueous zinc cells: **a** charge/discharge curves and **b** XRD patterns of the discharged electrodes.

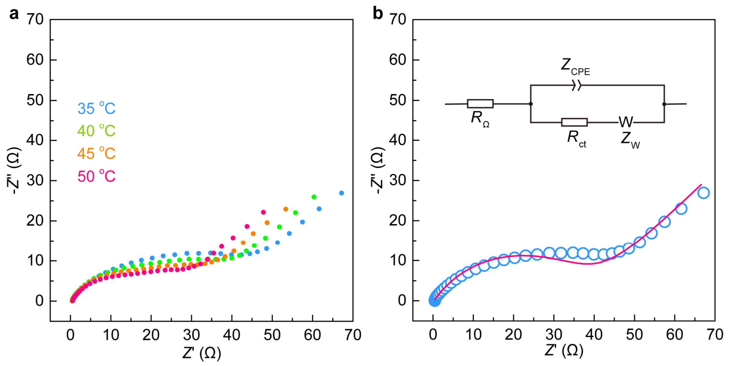

Supplementary Figure 14. EIS analysis of TABQ: **a** Nyquist plots at various temperatures and **b** the representative fitting of the 35 °C plot with a typical equivalent circuit (inset).

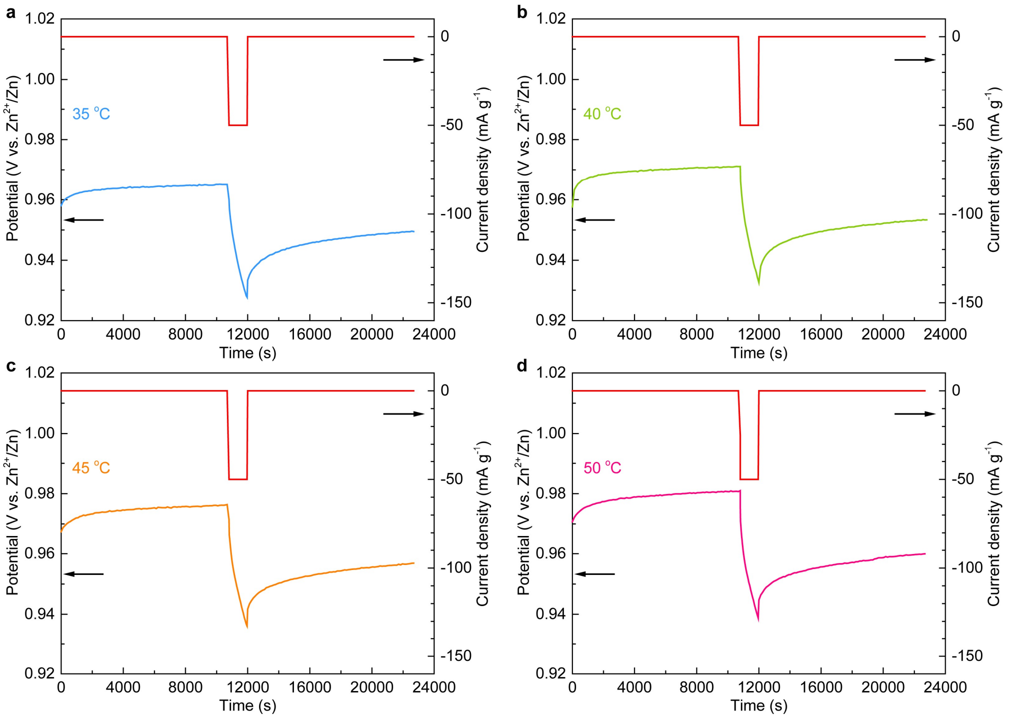

Supplementary Figure 15. GITT curves of TABQ at various temperatures.

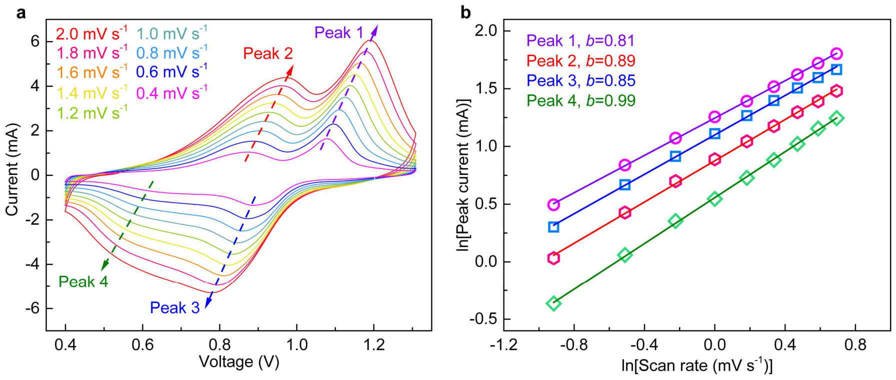

Supplementary Figure 16. Kinetics studies of TABQ: **a** CV curves at various scan rates; **b** the linear fits of $\ln(i)$ vs. $\ln(v)$ plots to calculate b values according to the equation of $i = av^b$.

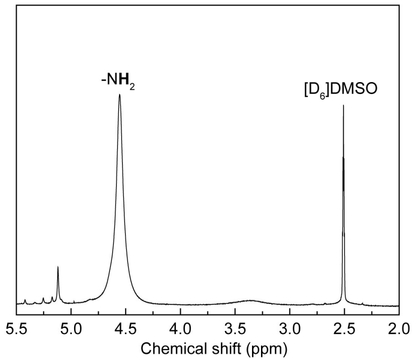

Supplementary Figure 17. ^{1}H NMR of the as-prepared TABQ.

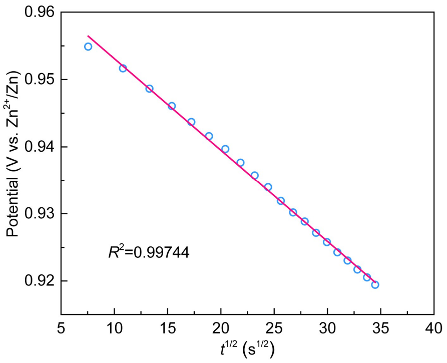

Supplementary Figure 18. The linear relationship between voltage and $t^{1/2}$ during GITT pulse.

Supplementary Table 1. Calculated R_{ct} of TABQ at various temperatures.

Temperature (°C)	R_{ct} (Ω)	Relative standard errors (%)
35	40.00	6.2
40	32.94	6.2
45	27.85	6.1
50	24.21	5.5

Supplementary Discussions.

Quantitative calculation of proton vs. Zn^{2+} storage in TABQ

In the discharged cathode, zinc exists in $Zn_4SO_4(OH)_6{\cdot}4H_2O$ as well as Zn^{2+} inserted TABQ, and SO_4^{2-} exists in $Zn_4SO_4(OH)_6{\cdot}4H_2O$.The weight percentage of zinc was measured by inductively coupled plasma optical emission spectroscopy (ICP-OES). The SO_4^{2-} was extracted from the discharged cathode with dilute hydrochloric acid, and the amount was measured by ion chromatography (IC). The ICP-OES and IC analysis resulted in Zn and SO_4^{2-} weight percentages of 17.34% and 5.76% in the discharged cathode, respectively.

The discharged cathode contains the mixture of: (1) proton inserted TABQ (2H-TABQ), (2) Zn^{2+} inserted TABQ (Zn-TABQ), (3) $Zn_4SO_4(OH)_6{\cdot}4H_2O$ and (4) KB and PVDF. Their weight percentages obey the following equations:

$$\text{2H-TABQ wt\%} + \text{Zn-TABQ wt\%} + Zn_4SO_4(OH)_6{\cdot}4H_2O\ \text{wt\%} + [\text{KB} + \text{PVDF}]\ \text{wt\%} = 100\% \quad \text{(S1)}$$

$$SO_4^{2-}\ \text{wt\%} = Zn_4SO_4(OH)_6{\cdot}4H_2O\ \text{wt\%} \times \omega[SO_4^{2-}\ \text{in}\ Zn_4SO_4(OH)_6{\cdot}4H_2O] = 5.76\% \quad \text{(S2)}$$

$$\text{Zn wt\%} = \text{Zn-TABQ wt\%} \times \omega[\text{Zn in Zn-TABQ}] + Zn_4SO_4(OH)_6{\cdot}4H_2O\ \text{wt\%} \times \omega[\text{Zn in}\ Zn_4SO_4(OH)_6{\cdot}4H_2O] = 17.34\% \quad \text{(S3)}$$

$$\text{TABQ wt\%} = \text{2H-TABQ wt\%} \times M_w[\text{TABQ}]/M_w[\text{2H-TABQ}] + \text{Zn-TABQ wt\%} \times M_w[\text{TABQ}]/M_w[\text{Zn-TABQ}] = [\text{KB} + \text{PVDF}]\ \text{wt\%} \quad \text{(S4)}$$

The meaning of the symbols in the above equations are as below:

2H-TABQ wt%: weight percentage of proton inserted TABQ in the discharged cathode;

Zn-TABQ wt%: weight percentage of Zn^{2+} inserted TABQ in the discharged cathode;

$Zn_4SO_4(OH)_6{\cdot}4H_2O$ wt%: weight percentage of $Zn_4SO_4(OH)_6{\cdot}4H_2O$ in the discharged cathode;

[KB + PVDF] wt%: weight percentage of KB and PVDF in the discharged cathode;

$\omega[SO_4^{2-}$ in $Zn_4SO_4(OH)_6{\cdot}4H_2O]$: mass fraction of SO_4^{2-} in $Zn_4SO_4(OH)_6{\cdot}4H_2O$, which is 18.07%;

ω[Zn in $Zn_4SO_4(OH)_6{\cdot}4H_2O$]: mass fraction of Zn in $Zn_4SO_4(OH)_6{\cdot}4H_2O$, which is 49.19%;

ω[Zn in Zn-TABQ]: mass fraction of Zn in Zn^{2+} inserted TABQ, which is 28.00%;

M_w[TABQ]: molecular weight of TABQ, which is 168.15 g mol^{-1};

M_w[2H-TABQ]: molecular weight of proton inserted TABQ, which is 170.17 g mol^{-1};

M_w[Zn-TABQ]: molecular weight of Zn^{2+} inserted TABQ, which is 233.54 g mol^{-1}.

The above equations result in 2H-TABQ wt% and Zn-TABQ wt% of 29.14% and 5.93%, respectively. Therefore, the mole of inserted proton is 13.5 times of Zn^{2+}. It confirms the domination of proton storage in TABQ during the redox processes.

Discussion of pH influence on TABQ redox potential

The potential difference between pH~0 and pH~4 electrolytes can be explained by Nernst shift as discussed below. The redox reaction on TABQ is written as:

$$\text{TABQ} + 2\text{H}^+ + 2\text{e}^- \rightleftharpoons 2\text{H-TABQ} \tag{S5}$$

The potential (E) is calculated according to the Nernst equation:

$$E = E^\circ + (0.0592/2)\lg(a_{\text{TABQ}} \times a_{\text{proton}}{}^2 / a_{\text{2H-TABQ}}) \tag{S6}$$

With both TABQ and 2H-TABQ in the solid state, the equation is rearranged to:

$$E = E^\circ + 0.0592\lg(a_{\text{proton}}) \tag{S7}$$

The proton activities in pH~4 and pH~0 electrolytes are around 4 and 0, respectively. Therefore,

$$E[\text{pH}\sim4] = E^\circ - 0.0592\times4 \tag{S8}$$

$$E[\text{pH}\sim0] = E^\circ - 0.0592\times0 \tag{S9}$$

The potential difference between the two groups of electrolytes is around:

$$E[\text{pH}\sim0] - E[\text{pH}\sim4] = 0.24 \text{ V} \tag{S10}$$

The value is close to the potential difference we obtained experimentally, confirming the proton involved redox reaction in TABQ.
